# Supplementary material for: CLEC12A Binds to Legionella pneumophila but Has No Impact on the Host’s Antibacterial Response
Source: Int J Mol Sci. 2023 Feb 15;24(4):3891. doi: 10.3390/ijms24043891 (PMC9967056; doi:10.3390/ijms24043891)
Supplement: Supplementary file 1 [file ijms-24-03891-s001.zip › ijms-2208110-supplementary.pdf]

**CLEC12A binds to *Legionella pneumophila* but does not significantly influence antibacterial host response**

Supplements

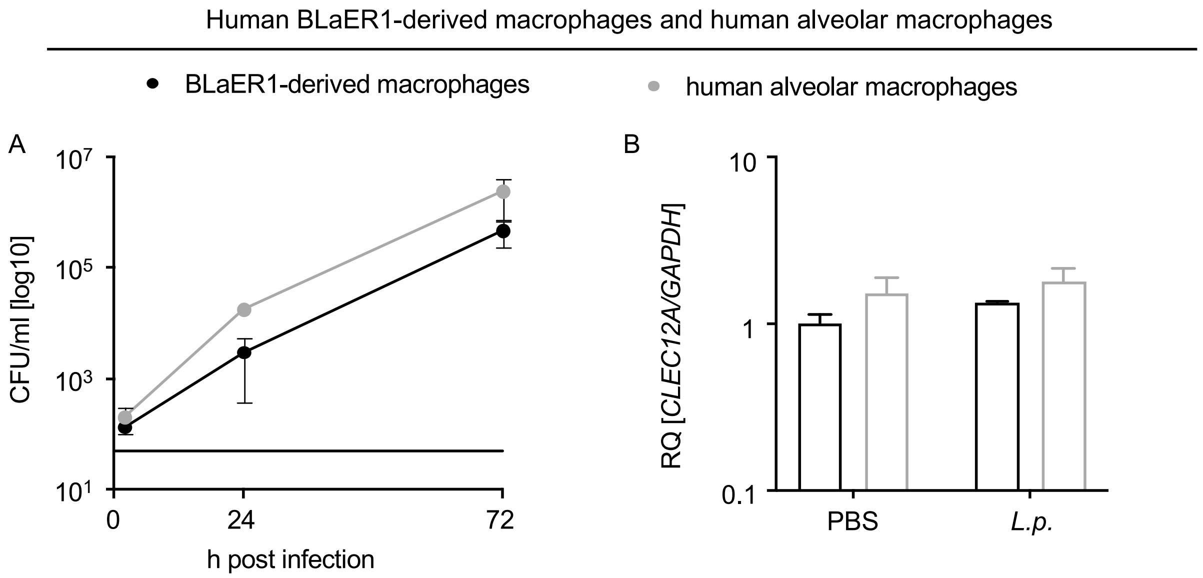


**Figure S1: Human BLaER1-derived macrophages support replication of *L. pneumophila* and show similar expression levels of *CLEC12A* as human alveolar macrophages.** (A) Human alveolar macrophages and BLaER1-derived human macrophage-like cells were infected with *L. pneumophila* JR32 WT ("*L.p.*") at a MOI of 0.1 and bacterial replication was evaluated over the course of 72 h. (B) Expression of *CLEC12A* in human alveolar macrophages and BLaER1 macrophages after infection with *L. pneumophila* at a MOI of 10 for 8 h or control treatment was assessed by qRT-PCR. Two independent experiments were carried out in triplicates. All data represent the mean ± SD of one representative experiment.
